# Overexpression of RKIP Inhibits Cell Invasion in Glioma Cell Lines through Upregulation of miR-98

**DOI:** 10.1155/2013/695179

**Published:** 2013-12-12

**Authors:** Zigui Chen, Quan Cheng, Zhiming Ma, Haipeng Xi, Renjun Peng, Bing Jiang

**Affiliations:** Department of Neurosurgery, Xiangya Hospital, Central South University, Changsha, Hunan 410011, China

## Abstract

Raf-1 kinase inhibitor protein (RKIP) is a tumor and metastasis suppressor in cancer cells. MicroRNAs (miRNAs) have been suggested to play a vital role in tumor initiation and progression by negatively regulating oncogenes and tumor suppressors. Quite recently, studies have identified some miRNAs operating to promote or suppress tumor invasion or metastasis via regulating metastasis-related genes, providing potential therapeutic targets on antimetastasis strategy. In this study, we found that the expression of RKIP and miR-98 in glioma tissues were significantly lower than that in normal brain tissues. Overexpression of RKIP upregulated miR-98 expression and inhibited glioma cell invasion and miR-98 target gene HMGA2 but had no effect in glioma cell proliferation. Moreover, forced expression of miR-98 accelerated the inhibition of glioma cell invasion and the expression of HMGA2 also had no effect in glioma cell proliferation. Our findings newly described RKIP/miR-98 to HMGA2 link and provided a potential mechanism for glioma cell invasion. RKIP and miR-98 may illustrate the potential therapeutic utility of signaling pathway signatures.

## 1. Introduction

Gliomas are the most common primary brain tumors [1]. Ras signaling was found to be required for the maintenance of glioma tumor growth in vivo [2]. Recent results indicate that Ras/Raf/MAPK pathway activation in glioma is achieved much more frequently by copy number gains than by mutations [3]. Interestingly, a Ras inhibitor can block both glioma cell migration and anchorage-independent proliferation [4]. Furthermore, a combination of Raf and mTOR inhibitors reduces glioma cell proliferation and invasion [5]. Raf kinase inhibitory protein (RKIP), also known as phosphatidylethanolamine binding protein, is involved in regulation of growth and differentiation of mammalian cells by inhibiting Raf and thereby negatively regulating growth factor signaling by the Ras/Raf/MAPK signal transduction pathway [6–8]. Lack of RKIP has been shown to promote tumor progression in a variety of human cancers [7].

A recent report has identified the critical role of RKIP in induction of let-7/miR-98. RKIP represses invasion, intravasation, and bone metastasis of breast tumor cells through a signaling cascade involving inhibition of MAPK, Myc, and LIN28 which leads to induction of the microRNA let-7/miR-98 and downregulation of its target genes [9, 10]. Nevertheless, the biological link of RKIP/miR-98 in the malignant progression of gliomas remains to be elucidated.

High mobility group protein A2 (high mobility group A2, (HMGA2)) as one of miR-98 target genes [11] is a recently discovered nonhistone chromatin protein, which is closely related to tumorigenesis, invasion, and metastasis of tumors, which have high extent and levels of expression in epithelial or interstitial malignant and are dependent on the metastasis of malignant and have poor prognosis [12–17].

In the present study, we confirmed the regulatory relationship between RKIP,  an antioncogene and a known tumor suppressive miRNA, and miR-98. We provided lines of evidences that over-expression of RKIP could inhibit glioma cell invasion at least partly through upregulation of miR-98.

## 2. Materials and Methods

### 2.1. Human Tissue Samples

All human normal brain and glioma tissue samples were obtained from the Department of Neurosurgery, Xiangya Hospital Central-South University. This study procedure was approved by The Institutional Review Board at the hospital. All participants provided written informed consent. Tissue samples were collected during surgery. For each sample, the major portion of tissue was frozen immediately in liquid nitrogen for molecular analysis, and the remaining tissue was fixed in paraformaldehyde for histological examination. All samples were histologically classified and graded according to WHO guidelines by a clinical pathologist, were prepared for cases in the institute biorepository, and classified and selected based on diagnosis.

## 3. Cell Lines and Cell Transfection

The human glial cell HEB and three human glioma cell lines, including U251, U87, and SHG44, were purchased from American Type Culture Collection. Cells were grown routinely in RPMI-1640 medium (Invitrogen, CA, USA) supplemented with 10% fetal bovine serum (Gibco, CA, USA) and cultured in a 37°C humidified atmosphere of 5% CO_2_. Ectopic expression of RKIP in cells was achieved by transfection with RKIP ORF clone (Neuron Bioscience, Shanghai, China) using Lipofectamine 2000 (Invitrogen, CA, USA). Overexpression of miR-98 was performed using pri-miR-98 (Neuron Bioscience, Shanghai, China). Cells were plated in 6-well clusters or 96-well plates and transfected for 24 h or 48 h. Transfected cells were used in further assays or RNA/protein extraction.

### 3.1. RNA Extraction and SYBR Green Quantitative PCR Analysis

Total RNA was extracted from cells using Trizol reagent (Invitrogen, CA, USA). Mature miR-98 expressions in cells were detected using a Hairpin-it TM miRNAs qPCR kit (Genepharma, Shanghai, China). Expression of RNU6B was used as an endogenous control. RKIP expression was measured by SYBR green qPCR assay (Takara, Dalian, China). Data were processed using 2^−ΔΔCT^ method.

### 3.2. Luciferase Assay

U251 and U87 cells were seeded into a 24-well plate. After being cultured overnight, cells were co-transfected with the wild-type and mutated HMGA2 3′UTR reporter plasmid and pRL-TK plasmids or transfected with miR-98 and miR-scrambled control precursors (miR-SCR). Luciferase assays were performed 48 h after transfection using the Dual Luciferase Reporter Assay System (Promega, WI, USA).

### 3.3. Western Blot Analysis

Immunoblotting was performed to detect the expression of RKIP and HMGA2 in glioma cell lines. Cultured or transfected cells were lysed in RIPA buffer with 1% PMSF. Protein was loaded onto a SDS-PAGE minigel and transferred onto PVDF membrane. After probed with 1 : 1000 diluted rabbit polyclonal RKIP and HMGA2 antibody (Abcam, MA, USA) at 4°C overnight, the blots were subsequently incubated with HRP-conjugated secondary antibody (1 : 5000). Signals were visualized using ECL substrates (Millipore, MA, USA). B-Actin was used as an endogenous protein for normalization.

### 3.4. BrdU Incorporation Assay

DNA synthesis in proliferating cells was determined by measuring 5-bromo-2-deoxyuridine (BrdU) incorporation. BrdU assays were performed at 24 h and 48 h after transfecting U251 or U87 cells with RKIP or control vector. The transfected cells were seeded in 96-well culture plates at a density of 2 × 10^3^ cells/well, cultured for 24 h or 48 h, and incubated with a final concentration of 10 *μ*M BrdU (BD Pharmingen, San Diego, CA, USA) for 2 h to 24 h. At the end of the incubation period, the medium was removed, the cells were fixed for 30 min at RT, incubated with peroxidase-coupled anti-BrdU antibody (Sigma-Aldrich) for 60 min at RT, washed three times with PBS, and incubated with peroxidase substrate (tetramethylbenzidine) for 30 min, and the absorbance values were measured at 490 nm. Background BrdU immunofluorescence was determined in cells not exposed to BrdU but stained with the BrdU antibody.

### 3.5. Cell Invasion Assay

**Figure 1:**
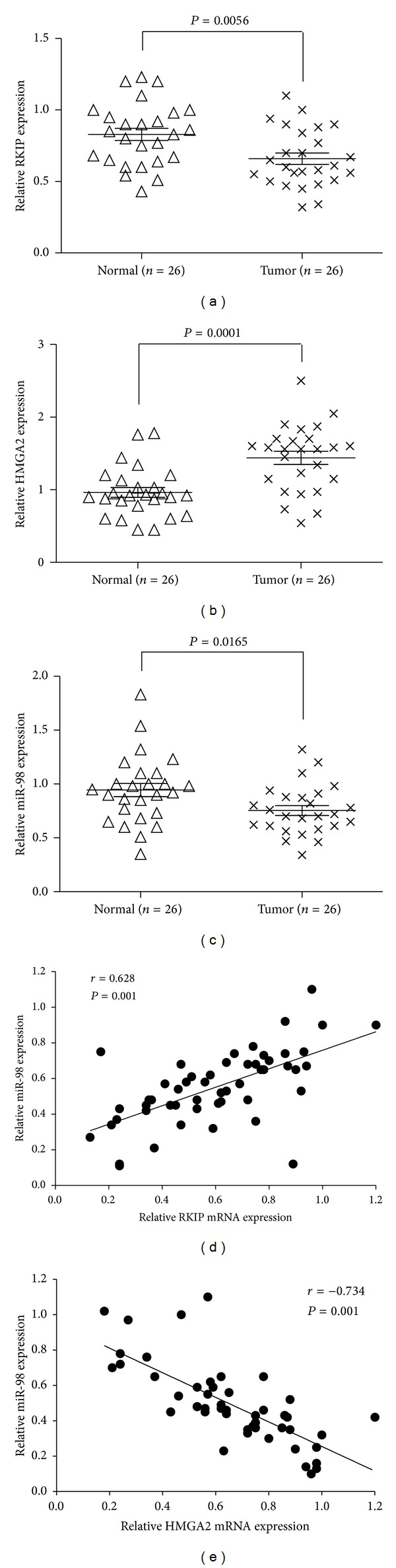
The miR-98 levels were positively correlated with the RKIP mRNA levels and negatively correlated with the HMGA2 mRNA levels in gliomas tissues. ((a), (b), (c)) The expression of RKIP mRNA, HMGA2 mRNA, and miR-98 was tested by quantitative RT-PCR in gliomas tissues compared to the adjacent normal brain tissues in a panel of matched tissues from 26 glioma patients (Wilcoxon's paired test, *P* values shown in the figures). ((d), (e)) Dot plots represent RKIP (HMGA2) mRNA relative expression level against miR-98 relative expression level. The lines represent approximated curves. The correlation coefficient (*r*) and the *P* value indicate the statistical significance of the negative correlation between the *x* and *y* variables. Results showed that the expression of miR-98 was positively correlated with RKIP mRNA and negatively correlated with HMGA2 mRNA. The figure is representative of three experiments with similar results.

The invasive potential of cells was evaluated using transwell inserts with 8 *μ*m pores (Coring, NY, USA). For invasion assay, at 24 h after transfection, 2.0 × 10^5^ cells (for Figure 6) and 1 × 10^6^ cells (for Figure 7) in serum free medium were added to each upper insert pre-coated with matrigel matrix (BD, NJ, USA). 500 *μ*L 10% FBS medium was added to the matched lower chamber. After 48 h incubation, noninvaded cells were removed from the upper surface of the Transwell membrane with a cotton swab, and invaded cells on the lower membrane surface were fixed in methanol, stained with 0.1% crystal violet, photographed, and counted. Inserts were conducted in triplicate in three separate experiments.

### 3.6. Statistical Analysis

All data from 3 independent experiments were expressed as mean ± SD and processed using SPSS17.0 statistical software. The expression of RKIP and miR-98 in glioma tissues and paired adjacent normal glial tissues was compared by Wilcoxon's paired test. A *P* value of <0.05 was considered to be statistically significant.

## 4. Results

### 4.1. The miR-98 Levels Were Positively Correlated with the RKIP mRNA Levels and Negatively Correlated with the HMGA2 mRNA Levels in Glioma Tissues and Cell Lines

**Figure 2:**
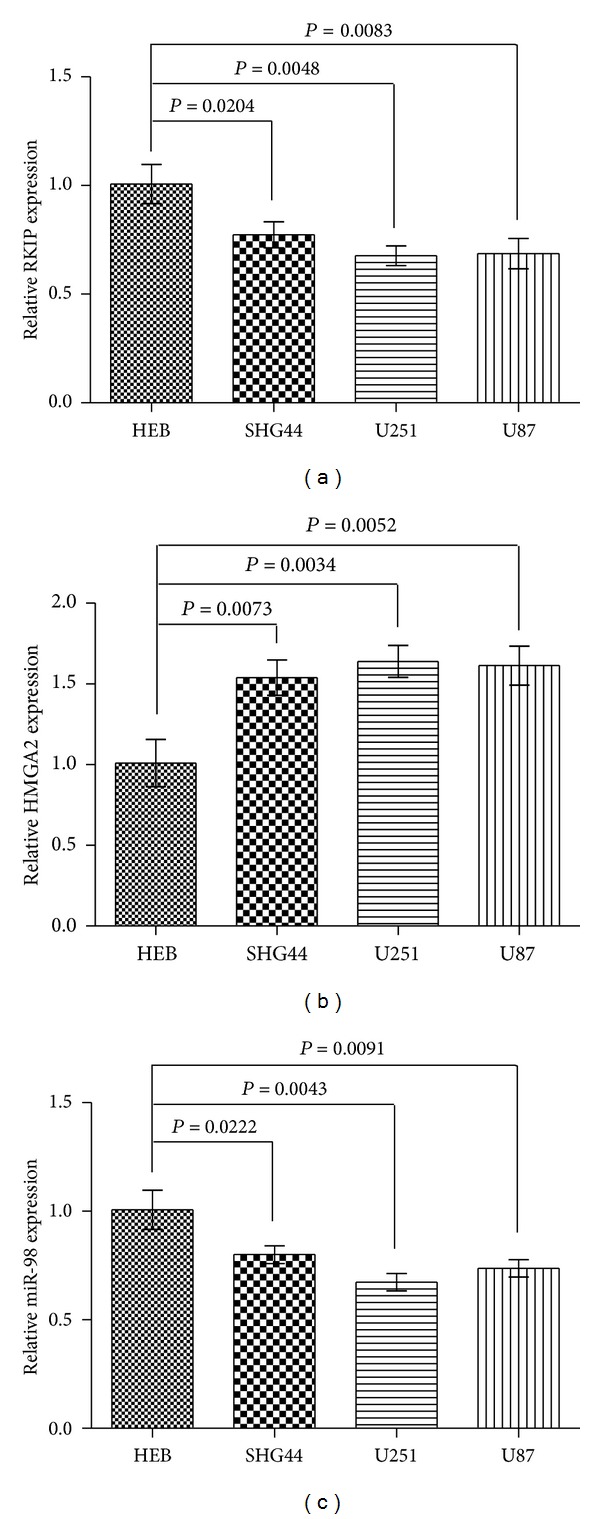
The RKIP mRNA levels, HMGA2 mRNA levels and miR-98 levels in glioma cells. ((a), (c)) Decreased expression of RKIP mRNA and miR-98 was tested by quantitative RT-PCR in glioma cells compared to the human glial cell HEB (Wilcoxon's paired test, *P* values shown in the figures). (b) Increased expression of HMGA2 was tested by quantitative RT-PCR in glioma cells compared to the human glial cell HEB (Wilcoxon's paired test, *P* values shown in the figures). The figure is representative of three experiments with similar results.

We performed SYBR green quantitative PCR analysis to detect the expression level of RKIP, HMGA2, and miR-98 in glioma tissues and cell lines. In the large panel of 26 cases of primary glioma tissues and their adjacent normal glial tissues, our results showed that miR-98 was significantly decreased in 21 (81%) in glioma tissues and RKIP in 19 (73%) and HGMA2 increased in 22 (85%) when compared with that in the paired adjacent normal tissues (Figures 1(a), 1(b), and 1(c)). Moreover, the expression of miR-98 was positively correlated with RKIP relative expression and negatively correlated with HMGA2 in tumor tissues (Figures 1(d) and 1(e)). In addition, we extended our test to one human glial cell and three human glioma cell lines. The total three cell lines showed a notable low expression of miR-98 and RKIP and high expression of HMGA2, whereas the control human glial cell expressed a strong level of it (Figures 2(a)–2(c)). These results suggested miR-98 levels positively correlated to the levels of RKIP expression and negatively correlated to the levels of HMGA2 expression in glioma tissues and cell lines.

### 4.2. miR-98 Directly Targets HMGA2

**Figure 3:**
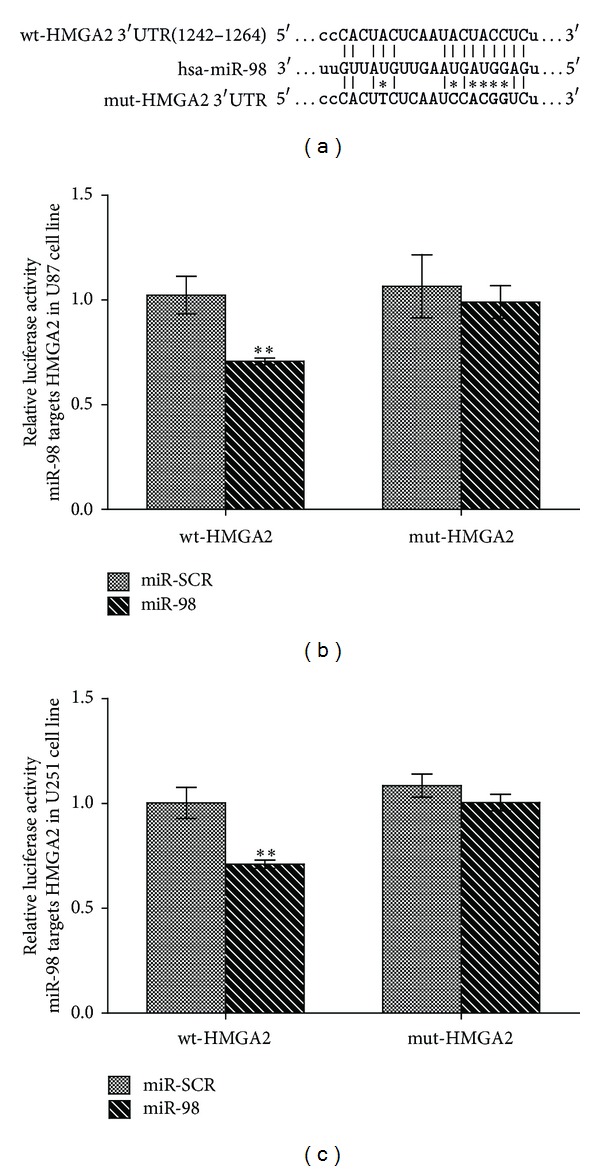
miR-98 directly targets HMGA2 by binding to its 3′UTR. (a) The predicted miR-98 binding site within HMGA2 3′UTR and its mutated version by site mutagenesis are as shown. (b) The repression of luciferase activity by HMGA2 3′UTR was dependent on miR-98 both in U251 and U87. Mutated HMGA2 3′UTR abrogated miR-98 mediated repression luciferase activity (**P < 0.01).

The combining sites of HMGA2 3′UTR with miR-98 were predicted by TargetScan and microRNA.org. A sequential replacement of a 6-base pair region was performed to produce mutant vector. (Figure 3(a)). To further investigate if the predicted binding site of miR-98 to 3′UTR of HMGA2 is responsible for this regulation, we cloned the 3′UTR of HMGA2 downstream to a luciferase reporter gene (wt-HMGA2); its mutant version (mut-HMGA2) by the binding site mutagenesis was also constructed. We cotransfected wt-HMGA2 vector and miR-98 mimics or scramble control into U251 and U87 cells. The luciferase activity of miR-98 transfected cells was significantly reduced compared to scramble control cells (Figure 3(b)).

### 4.3. RKIP Inhibits HMGA2 Expression via miR-98 Signaling

**Figure 4:**
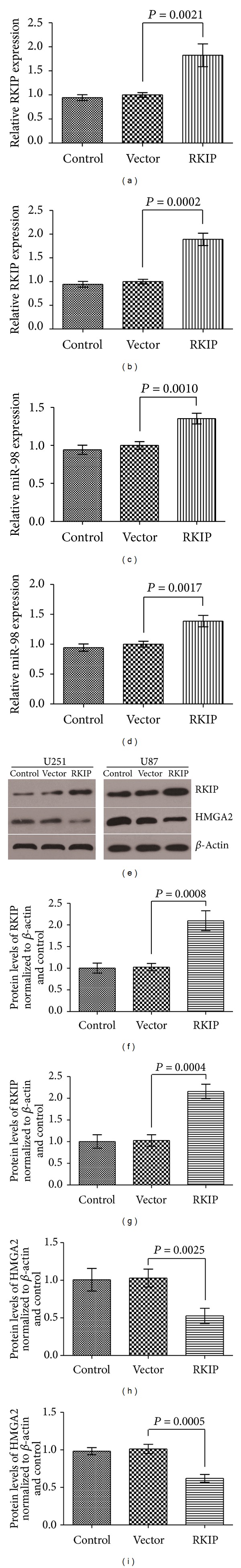
Force expression of RKIP enhances miR-98 expression and inhibits HMGA2 expression. U251 or U87 cells were stably infected with vector control and RKIP ORF clone. ((a), (b)) Quantitative RT-PCR quantification of RKIP mRNA or miR-98 expression compared to U251 or U87 cells transfected with vector control. (c) Western blot of RKIP protein expression. Expression of RKIP was forced, and expression of HMGA2 was inhibited by overexpression of RKIP, compared to U251 or U87 cells expressing vector. ((d), (e)) Band intensities were quantitated by Image-Pro Plus. The intensities of the bands corresponding to RKIP and HMGA2 were compared to those corresponding to *β*-actin and control untransfected U251 or U87. The figure is representative of three experiments with similar results.

**Figure 5:**
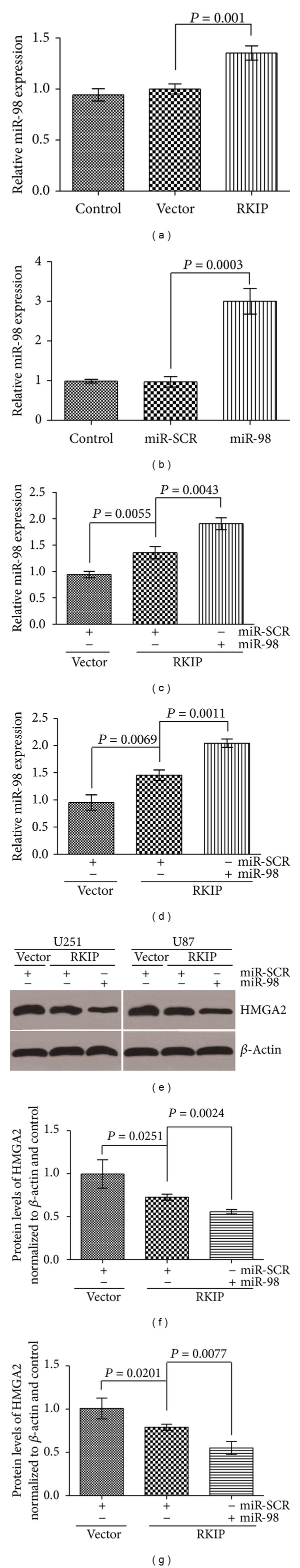
Overexpression of miR-98 enhances the effects of RKIP on miR-98 expression and HMGA2 expression. U251 or U87 cell line was cotransfected with RKIP ORF clone and miR-98 mimics. After 48 h, the level of miR-98 or HMGA2 protein was analyzed by quantitative RT-PCR or western blot. (a) Overexpression of miR-98 increased miR-98 expression. (b) Overexpression of miR-98 enhances the effects of RKIP on miR-98 expression. (c) Overexpression of miR-98 increased the effects of RKIP on HMGA2 protein expression. ((d), (e)) Band intensities were quantitated by Image-Pro Plus. The intensities of the bands corresponding to HMGA2 were compared to those corresponding to *β*-actin and control cotransfected with miR-SCR and pcDNA3.1 vector. The figure is representative of three experiments with similar results.

To further study the relationship of RKIP and miR-98, we transfected U251 and U87 cells with RKIP ORF clone. Quantitative RT-PCR showed that, at 72 h after transfection, the expression of miR-98 and RKIP was upregulated as compared with vector (Figures 4(a), 4(b), 4(c), and 4(d)) both in U251 and U87. Moreover, we observed the enhanced RKIP in the two cells significantly repressed HMGA2 protein expression compared to cells transfected with vector control by western blot (Figures 4(c) and 4(e)). Meanwhile, we forced the two cells overexpression of miR-98 by transfecting with miR-98 mimics (Figure 5(a)). When we co-transfected with RKIP ORF clone and miR-98 mimics in the two cells, more apparent up-regulation of miR-98 relative expression was observed by quantitative RT-PCR (Figure 5(b)), and more significant inhibition of HMGA2 protein expression was tested by western blot assay (Figures 5(c) and 5(d)). These data suggested that a potential regulation of miR-98 by RKIP and RKIP might inhibit HMGA2 expression via miR-98 signaling.

### 4.4. Effect of RKIP/miR-98 Axis on Glioma Cells Proliferation and Invasion

**Figure 6:**
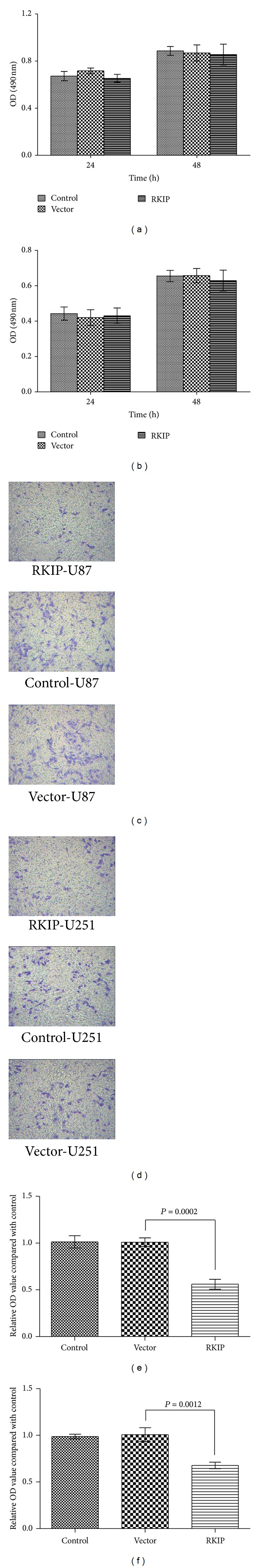
Effects of RKIP on the proliferation and invasion of U251 and U87 cell lines. U251 or U87 cell line was transfected with vector control and RKIP ORF clone. (a) BrdU cell proliferation assay. Ectopic overexpression of RKIP had no effect on proliferation of U251 or U87 cell line, compared to vector ((b), (c)) Transwell assay; upregulated RKIP expression significantly inhibited the invasion ability of U251 and U87 cell lines. The figure is representative of three experiments with similar results.

**Figure 7:**
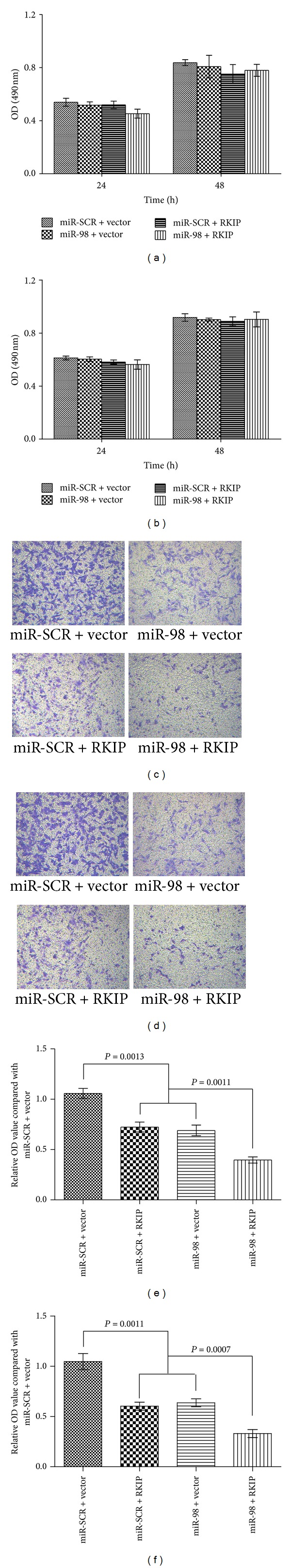
Overexpression of miR-98 enhances effects of RKIP on the proliferation and invasion of U251 or U87 cell line. U251 and U87 cells were cotransfected with RKIP ORF clone and miR-98. After 48 h, the proliferation of U251 or U87 was analyzed by BrdU assay and the invasion ability was observed by Transwell assay. (a) BrdU cell proliferation assay. Overexpression of miR-98 had no effects on the proliferation of U251 or U87 cell line. (b) More significant inhibition of invasion ability was observed by Transwell assay. The figure is representative of three experiments with similar results.

To validate if RKIP regulates glioma cells growth and invasion, we performed a proliferation assay (BrdU Assay) by transfecting RKIP or vector control into U251 and U87 cells. It showed that over-expression of RKIP had no effect on cell growth (Figure 6(a)). As shown in Figure 6(b), compared to the vector control, RKIP ORF clone transfected into U251 or U87 cells exhibited significant inhibition of invasion ability (Figures 6(b) and 6(c)). On the other hand, RKIP ORF clone and miR-98 mimics co-transfected into the two glioma cells; also, no proliferation of the cells was observed (Figure 7(a)), but more significant inhibition of invasion ability was tested by Transwell assay (Figures 7(b) and 7(c)). These results indicated that RKIP functions as a potent tumor invasion repression gene through regulating miR-98 expression.

## 5. Discussion

Diffusely infiltrating gliomas are one of the most devastating cancers because they often show locally aggressive behavior and cannot be cured by existing therapies [18]. Like cancer in general, gliomas develop as a result of genetic alterations that accumulate throughout tumor progression [1, 19, 20]. Therefore, the elucidation of these molecular mechanisms, in particular the ones associated with cellular migration and invasion is crucial for a better prediction of glioma patients outcome and response to therapies [21].

RKIP is widely expressed in normal human tissues and has been studied for several years as an important regulator of several physiologic processes [22]. In addition, it is an important regulator of tumor cell invasion and metastasis [22–24]. Furthermore, it was reported to be a prognostic biomarker for a number of tumors including prostate, colorectal, GISTs, gastric adenocarcinoma of the intestinal subtype, hepatocellular carcinoma, pancreatic ductal adenocarcinoma and also in high grade gliomas [25–30]. miR-98 belongs to the mature let-7 family of miRNAs [31] and was initially found to be down regulated in leukemia cell lines [32]. Subsequent studies showed that the expression of miR-98 was also significantly decreased in solid tumors such as nasopharyngeal carcinoma and head and neck squamous cell carcinoma [33, 34]. In this study, we found the expression of RKIP and miR-98 in glioma tissues was significantly lower and HMGA2 was higher than that in normal brain tissues. These findings showed that miR-98 and HMGA2 might participate in regulating tumor cell invasion like RKIP.

On the other hand, over-expression of RKIP up-regulated miR-98 expression and inhibited glioma cell invasion but had no effect on glioma cell proliferation. How does RKIP regulate miR-98? miR-98 expression can be controlled at multiple levels, including synthesis of the primary transcript, Drosha processing to the precursor, and Dicer processing to the mature form [35]. Analysis of the miR-98 primary transcript by qRT–PCR showed an increase in response to RKIP, indicating that regulation occurs subsequent to primary transcription.

Our present findings are in accordance with previous reports in other types of tumors, where RKIP seems to be more important in migration of the cells, instead of as a proliferation suppressor [36–38]. Additionally, we find that RKIP inhibits miR-98 target gene HMGA2 which enhances invasion and regulates a number of target genes that contribute to invasion and metastasis in the glioma cell lines. Moreover, forced expression of miR-98 accelerated the inhibition of glioma cell invasion, and the expression of HMGA2 also had no effect in glioma cell proliferation. These results suggested that the RKIP/miR-98 to HMGA2 axis might play an important role in inhibiting glioma invasion and metastasis.

The RKIP/miR-98 to HMGA2 axis likely identifies a subpopulation of high-risk human gliomas by revealing a cellular signaling environment that is favourable to metastatic progression. Similar to other tumour suppressors, RKIP loss alone is not sufficient to promote invasion and metastasis unless RKIP depletion occurs in certain cellular signaling contexts. Finally, it is likely that the miR-98 pathway is one of the mechanisms by which RKIP regulates tumor cell invasion. Detailed investigation of genes that comprise the RKIP network should yield further insight into the mechanism by which RKIP suppresses metastatic progression. In conclusion, we newly described RKIP/miR-98/HGMA2 link and provided a potential mechanism for RKIP over-expression and contribution to gliomas invasion but not proliferation. On the other hand, restoration of miR-98 expression could have an important implication for the clinical management of gliomas.
